# Magnetic Platelets as a Platform for Drug Delivery and Cell Trapping

**DOI:** 10.3390/pharmaceutics15010214

**Published:** 2023-01-07

**Authors:** Oksana A. Mayorova, Olga I. Gusliakova, Ekaterina S. Prikhozhdenko, Roman A. Verkhovskii, Daniil N. Bratashov

**Affiliations:** 1 Science Medical Center, Saratov State University, 83 Astrakhanskaya Str., 410012 Saratov, Russia; 2 Department of General Educations, Saratov State Vavilov Agrarian University, 1 Theater Square, 410012 Saratov, Russia

**Keywords:** platelet, biocontainers, magnetite nanoparticles

## Abstract

The possibility of using magnetically labeled blood cells as carriers is a novel approach in targeted drug-delivery systems, potentially allowing for improved bloodstream delivery strategies. Blood cells already meet the requirements of biocompatibility, safety from clotting and blockage of small vessels. It would solve the important problem of the patient’s immune response to embedded foreign carriers. The high efficiency of platelet loading makes them promising research objects for the development of personalized drug-delivery systems. We are developing a new approach to use platelets decorated with magnetic nanoparticles as a targeted drug-delivery system, with a focus on bloodstream delivery. Platelets are non-nuclear blood cells and are of great importance in the pathogenesis of blood-clotting disorders. In addition, platelets are able to attach to circulating tumor cells. In this article, we studied the effect of platelets labeled with BSA-modified magnetic nanoparticles on healthy and cancer cells. This opens up broad prospects for future research based on the delivery of specific active substances by this method.

## 1. Introduction

The development of new drug-delivery systems with a high loading capacity and low side effects is currently an urgent task. The systemic administration of micro- and nanoscale containers loaded with an active substance and their passive or active targeting reduce the toxic effects on healthy tissues and the organism in general [1,2,3,4,5]. Targeted drug delivery provides the local rise in active substance concentration in the desired area that allows for achieving the positive therapeutic effect at the injection of a lower amount of drug and consequently decreases systemic side effects [6]. For these purposes, drug carriers based on various nanomaterials are being actively developed and studied. They include nanoparticles [7,8,9], polymer micelles, liposomes, and polyelectrolyte microcapsules [10,11]. However, the body has an innate immune defense system, which quickly recognizes and destroys foreign objects passed into the organism. Thus, systemically administered nanocarriers can be recognized as a target by the immune cells and internalized by phagocytes before the carriers reach the region of interest. In addition, significant disadvantages of many of these approaches are toxicity and limited biodegradability [12,13]. In this regard, biological drug carriers, such as erythrocytes, exosomes, and albumin, are currently being widely studied. Such biological containers have many advantages over synthetic delivery systems: no unwanted physiological response, good biodegradability, and long circulation time [14,15].

The first type of biological drug carriers is blood cells. Biocarriers based on blood cells are able to bypass the immune barrier, which makes them attractive objects for creating biological systems for targeted drug delivery in the field of personalized therapy [16]. At present, erythrocytes are the most comprehensively investigated blood-cells-based carriers. Erythrocytes have been proposed for the delivery of a chemotherapeutic agent, and the process of its encapsulation in erythrocytes has been studied [17]. Erythrocytes have also been considered as a container for immunosuppressive agents, the systemic administration of which can significantly improve the outcome of organ transplantation [18]. Moreover, thrombolytic agents have been obtained for the treatment of a wide range of diseases [19]. However, conventional approaches to the use of erythrocytes as drug carriers lead either to irreversible damage to the membrane or to the loss of RBC plasticity and invisibility to immune cells [20].

The second type of carriers is exosomes. Exosomes are able to deliver various types of cargo to the target cell by modifying the vesicular membrane or by direct encapsulation. Modified exosomes isolated from different cell types are able to interact site specifically with donor cells, thereby increasing their penetration into body tissues [21,22,23]. Despite all the advantages of using vesicular delivery systems in the production of exosomes, many critical problems remain, such as the lack of standardized production and purification. More research is needed on the source of the cell type for exosome formation, which can significantly affect the targeting and biological properties of carriers [24].

The third drug-delivery approach is based on serum albumin, which is a major protein of plasma. One of its functions is the delivery of small insoluble substances. The ability of plasma proteins to accumulate in tumor tissues due to their internalization by tumor cells underlies the development of targeted delivery systems. The high degree of albumin–drug interaction allows the desired drug to be delivered to the area of interest. The potential of albumin nanoparticles with encapsulated antitumor drugs for the treatment of various types of cancer both in vitro and in vivo is being studied. There is evidence of clinical trials of albumin-bound methotrexate in xenotransplantation of human tumors [25]. However, the unpredictability of the process of cross-linking albumin with the drug can lead to a violation of the conformational structure of the protein, and, as a result, the accumulation of conjugates not in the region of interest [26].

One of the promising approaches to the development of biological targeted delivery systems is based on the use of patient’s blood platelets. The main purpose of platelets is to prevent blood loss at sites of vascular damage. Platelets come into contact with the damaged surface, as a result of which they are activated: platelets turn into spherocytes. The appearance of spherocytes ensures the rapid contact of individual platelets with each other, slowing down the blood flow at the site of their activation [27]. In addition, the formation of a large amount of thrombotic masses (thrombophlebitis) can serve as an indirect confirmation of the presence of cancer tumors. It has been proven that tumor cells, including metastatic ones, increase the number of platelets, which in turn protect circulating tumor cells from the body’s immune response. As a consequence, an increased number of platelets promotes tumor growth and facilitates the transfer of metastatic cells to secondary lesions [28]. This platelet property has been used to develop doxorubicin targeting systems for the treatment of lymphoma [29]. S. Sarkar and coauthors showed that the platelet loading of doxorubicin reduces the cardiotoxicity of the encapsulated drug and inhibits the proliferation of Raj’s lymphoma cells [30]. A high loading capacity of platelets with doxorubicin without significant morphofunctional changes was also demonstrated in that article. The synergy of these qualities allows the use of patient’s own platelets as personalized drug-delivery systems.

In this study, the possibility of interaction of platelets with magnetic nanoparticles was investigated, and the functional ability of magnetic-containing platelets was evaluated. A comparison of the in vitro interaction between platelets containing magnetic nanoparticles and mouse normal and cancerous adherent cells (L-929, B16F10, respectively) or human normal and cancerous suspension cells (PBMC, THP-1, respectively) was carried out. This can open a broad avenue to using platelets as drug carriers for individual targeted therapy of diseases with reduced side effects compared to other types of carriers.

## 2. Materials and Methods

### 2.1. Materials

MEM, DMEM, RPMI-1640, Fetal Bovine Serum, 2-Methylmercaptoethanol were obtained from Gibco (Paisley, UK). Hoechst 33342 was obtained from Invitrogen (Waltham, MS, USA). N-(3-Dimethylaminopropyl)-N${}^{\prime }$-ethylcarbodiimide, Histopaque-1077, Calcein AM, glutaraldehyde, Nile red, Iron (III) chloride hexahydrate (99.8%), Iron (II) chloride tetrahydrate (99.8%), sodium hydroxide (99.8%), and citric acid (99.8%) were purchased from Sigma Aldrich (Steinheim, Germany). Hexamethyldisilizane (CAS 999-97-3) was purchased from EKOS-1 (Moscow, Russia). Sodium chloride (Ph. Eur., pure, pharma grade) was obtained from PanReac AppliChem (Darmstadt, Germany). All reagents were used without preliminary purification.

Millipore Milli Q water (18.2 MW·cm${}^{-1}$) from the Direct 8 system was used as an aqueous medium during all experiments.

### 2.2. Synthesis of Citrate-Stabilized Magnetite Nanoparticles

Magnetic nanoparticles were obtained by chemical precipitation from Fe(II) and Fe(III) salt solutions in basic media, as described by German et al. [31]. The mixing of the reagents and the washing steps were carried out in a nitrogen atmosphere in a chemical reactor CR-1 (Tetraquant, Moscow, Russia). The measurements of ζ-potential and size distribution of nanoparticles were performed using a Zetasizer Nano-ZS instrument (Malvern Instruments Ltd., Malvern, UK). The ζ-potential of the nanoparticles in aqueous suspension at pH 6.9 was −16.9±4 mV. The average size of the nanoparticles was 12±3 nm. The concentration of the magnetite colloid was 1.0 mg/mL.

### 2.3. Synthesis of FITC-Labeled and TRITC-Labeled BSA

First, 160 mg of BSA were dissolved in 40 mL of 0.1 M PBS buffer (pH 8). Afterward, the solutions of FITC in ethanol (5 mg/mL) or TRITC in DMSO (5 mg/mL) were prepared. Then, 40 mL of BSA solution was added to 5 mL of FITC/TRITC solution under gentle stirring and further the mixture was stirred under 4 °C in the dark for 12 h. Finally, freshly prepared FITC-conjugated (or TRITC-conjugated) BSA was dialyzed for 3 days in deionized water then freeze-dried 24 h after the cooling period using FreeZone Freeze Dryers Labconco (Kansas City, MO, USA).

### 2.4. Synthesis of Magnetite Nanoparticles Conjugated with BSA, BSA (FITC) or BSA (TRITC)

For the adsorption of either BSA, BSA (FITC) or BSA (TRITC) on the FeNP surface, 4.0 mL of BSA/BSA (FITC)/BSA (TRITC) (2 mg/mL), respectively, in 0.15 M NaCl was mixing with 8 mL of citrate-modified nanoparticles (pretreated with 0.3 M sodium citrate), which were sonicated for one minute. For the stabilization of the particles, 4 mg N-(3-dimethylaminopropyl)-N${}^{\prime }$-ethylcarbodiimide was added for cross-linking under vigorous stirring at room temperature for 4 h. Then, this suspension was shaken gently at room temperature for 4 h. Finally, FeNPs labeled with BSA/BSA (FITC)/BSA (TRITC) were filtered via MF-Millipore™ Membrane Filter (5 μm pore size).

### 2.5. Determination of Size and ζ-Potential of FeNP-BSA(FITC)

The size and ζ-potential of the magnetic nanoparticles modified by BSA(FITC) were measured by the dynamic light scattering (DLS) using a Zetasizer Nano-ZS (Malvern Instruments, Worcestershire, UK). The resulting sizes and ζ-potentials are average values from 25 measurements.

### 2.6. Platelet Investigation

The use of human blood obtained from healthy volunteers including the informed consent procedure was approved by the ethics board at the Saratov State Medical University (Saratov, Russia). Obtaining fresh whole blood and subsequent human platelets investigations were performed using the authors’ own whole blood.

#### 2.6.1. Separation of Platelets from Whole Blood

Platelets were isolated from whole blood according to standard protocol [32]. Briefly, fresh whole blood drawn into a syringe with 3.2% sodium citrate anticoagulant (9:1) was transferred to a 15 mL sterile tube and centrifuged at 150× *g* for 15 min at room temperature to obtain the upper phase: platelet-rich plasma (PRP). Then PRP was carefully transferred to a 15 mL sterile tube, ACD-A anticoagulant buffer (6.25 g sodium citrate, 3.1 g citric acid, 3.4 g D-glucose in 250 mL H_2_O) was added 1/10 v.v., and the mixture was centrifuged at 900× *g* for 5 min. The supernatant, platelet-pure plasma (PPP), was discarded. The resulting platelets were resuspended in 5 mL HEPES-buffered Tyrode’s solution (pH 7.4, containing 60 μM bovine serum albumin (BSA), 5.6 mM glucose, 5 μM calcium chloride, and 5 μM magnesium chloride).

#### 2.6.2. Labeling of Platelets with Magnetic Particles

A suspension of platelets isolated from whole blood in HEPES-buffered Tyrode’s solution was incubated with magnetite nanoparticles conjugated with either BSA or BSA(FITC) solutions (1 mg/mL iron concentration) at a ratio of 10:1 for 30 min at 37 °C. Platelets were centrifuged to remove excess particles (900× *g*, 5 min, room temperature), resuspended in HEPES-buffered Tyrode’s solution.

#### 2.6.3. Magnetic Separation of Magnetite Labeled Platelets

A portion of platelets labeled with FeNP-BSA or FeNP-BSA(FITC) was separated from unlabeled ones by magnetic separation using the OctoMACS™ Separator system (Miltenyi Biotec GmbH, Teterow, Germany).

#### 2.6.4. Magnetite-Rich Platelet Staining

Staining of platelets rich in FeNP-BSA or FeNP-BSA(FITC) was performed by incubating a mixture of platelets with Nile Red solution (0.1 mg/mL in DMSO) at a ratio of 10:1 for 30 min at 37 °C. Stained platelets were centrifuged (900× *g*, 5 min, room temperature) and resuspended in HEPES-buffered Tyrode’s solution. During staining and washing, platelets were treated with ACD-A buffer (ADC-A: platelets, 1:10 ratio) to prevent activation.

#### 2.6.5. Platelet Aggregation Activity after Labeling

The retention of platelet aggregation activity containing magnetic nanoparticles was quantified according to a standard protocol [33]. ADP with an initial concentration of 1 mM (0.5 mg/mL) was used as an inducer of platelet aggregation. Briefly, in a 96-well plate, 30 μL of ADP working solution (20, 40, 100, 200 μM) was added to 300 μL of FeNP-BSA labeled platelets. The resulting mixture was incubated for 10 min at 37 °C with orbital agitation (200 rpm). The optical density value was measured using a multifunctional hybrid photometer Synergy H1 (BioTek Instruments, Winooski, VT, USA) at a wavelength of 650 nm. Platelets which did not have FeNP-BSA were used as controls.

#### 2.6.6. Flow Cytometry

Positive (with FeNP-BSA(FITC)) and negative (without FeNP-BSA(FITC)) portions of platelets after incubation with FeNP-BSA(FITC) were counted and evaluated using the imaging flow cytometer Amnis ImageStream X Mk II (Luminex Corporation, Austin, TX, USA). Fluorescence was excited by a 488 nm laser at 50 mW power. Flow cytometry data were processed using IDEAS software (Luminex Corporation, Austin, TX, USA).

### 2.7. Isolation of PBMC from Whole Blood

Peripheral blood mononuclear cells (PBMC) were isolated from whole blood according to standard protocol implemented Histopaque-1077 as a separation medium. Briefly, the whole blood sample (4 mL) was diluted with RPMI-1640 culture medium (4 mL). Then, 4 mL of Histopaque-1077 was added to 15 mL tube. Diluted whole blood was gently layered on the top of the Histopaque-1077. The following centrifugation was performed at 400× *g* for 30 min with no brake. The resultant thin white ring between Histopaque-1077 and plasma was aspirated with a pastette and transferred to another 15 mL tube. The washing procedure was performed three times with DPBS (5 mL) and 5 min centrifugation at 800× *g*.

### 2.8. Cell Culturing

Mouse fibroblasts (L-929 cell line), mouse melanoma (B16F10 cell line), mouse colon carcinoma (CT26 cell line), human monocyte (THP-1 cell line), and primary human mononuclear cell (PBMC) were used for observation of cell interaction. L-929 was cultured in a minimum essential medium (MEM) supplemented with a 10% fetal bovine serum (FBS), B16F10 was cultured in Dulbecco’s modified Eagle medium (DMEM) with a 10% FBS, CT26 was cultured in the Roswell Park Memorial Institute (RPMI-1640) medium with a 10% FBS, THP-1 and PBMC were cultured in RPMI-1640 medium with a 10% FBS and 0.05 mM 2-methylmercaptoethanol in a humidified incubator containing 5% CO_2_ at 37 °C. All cells were cultured with 1% penicillin–streptomycin.

#### 2.8.1. Cell Interaction Study

Interaction between platelets and continuous cell line (L-929, B16F10, CT26, THP-1) or PBMC was observed during 24 h of incubation. Initially, cell lines (L-929, B16F10, CT26, THP-1, and PBMC) were seeded in Petri dishes at the density of 300,000 cells and incubated overnight. The following day, platelets-FeNP-BSA were added to Petri dishes (20 platelets per seeded cell) after the culture mediums were renewed in each cell line. Subsequently, cells were visualized with Leica TCS SP8 X inverted confocal microscope (Leica Microsystems, Wetzlar, Germany) during the first hour of observation at 37 °C and kept in an incubator (Innova CO-170, New Brunswick Scientific, Enfield, CT, USA) at 37 °C for 24 h before the next measurement with confocal laser scanning microscopy (CLSM).

#### 2.8.2. Confocal Laser Scanning Microscopy

Cells were stained with Calcein AM (cytoplasm) and Hoechst 33342 (nuclei) according to the manufacturer’s protocol. Briefly, the stock staining solution was made by mixing culture media DMEM without FBS (3 mL) and Hoechst 33342 (3 μL), and adding Calcein AM solution (3 μL for L-929, B16F10, CT26 cell lines; 0.5 μL for THP-1, PBMC). Platelets were stained with Nile red as described above. Cell imaging was performed during the first hour (every 5 min), at 2 and 24 h.

CLSM was performed with Leica TCS SP8 X inverted confocal microscope (Leica Microsystems, Wetzlar, Germany) equipped with diode (405 nm) and argon laser sources. All measurements were performed using 20×/0.70 N.A. objective. The following settings were used: 405 nm excitation with 415–477 nm detection (blue channel, Hoechst 33342, cell nuclei), 488 nm excitation with 500–550 nm detection (green channel, Calcein AM, cell cytoplasm), and 514 nm excitation with 605–700 nm detection (red channel, Nile red, platelets).

### 2.9. Cell Samples Preparation for SEM and TEM Morphological Study

Cell samples were fixed with 2.5% glutaraldehyde for 1 h in the refrigerator (+4 °C) after fluorescent microscopy. The dehydration procedure was carried out by a sample, holding in a series of rising ethanol concentrations in ultrapure water (50–100% with 10% step). The washing of samples after each step was performed three times with fresh DPBS. An appropriate drying procedure was provided by the use of hexamethyldisilizane (HMDS) to protect cell-surface structures against shrinkage and collapse.

#### 2.9.1. Scanning Electron Microscopy

For SEM imaging, thin glass that was a bottom from Petri dish (Eppendorf Cell Imaging Dishes with cover glass bottom, Eppendorf, Hamburg, Germany) with cells was placed on a silicon substrate to remove the extra charge and gold-sprayed. SEM measurements were performed with a MIRA II LMU (TESCAN, Brno, Czech Republic) microscope at an operating voltage of 30 kV. Images were observed at a 10,000× magnification.

The following cell samples were investigated: platelets with and without magnetite adsorption; L-929, B16F10, THP-1, and PBMC alone and after incubation with magnetite-rich platelets (24 h).

#### 2.9.2. Transmitted Electron Microscopy

For TEM imaging, the platelets were placed on a copper mesh coated with a formvar solution, and then the samples were sputtered with gold in vacuum. TEM measurements were performed with a MIRA II LMU (TESCAN, Brno, Czech Republic) microscope at an operating voltage of 30 kV. Images were observed at a 10,000× magnification.

SEM and TEM measurements were performed at the Educational and Scientific Institute of Nanostructures and Biosystems of the Saratov State University, Saratov, Russia.

### 2.10. Magnetic Capturing THP-1 Cells after Platelets Adhesion

THP-1 cells were stained with Calcein AM and Hoechst 33342 as described above and seeded to a 48-well plate at concentration 10${}^{6}$ cells per well (in 1 mL of complete culture media). Platelets-FeNPs-(BSA-TRITC) were added to 3 wells at concentration 20 platelets per THP-1 cell (experimental wells). Control THP-1 cells were left at another 3 wells (without co-cultivation with platelets). Co-cultivation lasted 24 h.

#### Estimation of Magnetic Capturing Efficiency by Flow Cytometry

Cells were carefully aspirated and transferred to 1.5 mL tubes after 24 h of incubation. Tubes containing THP-1 and platelets-FeNPs-(BSA-TRITC) were held against a magnet for 15 min. The cylindrically shaped permanent NdFeB magnet with diameter of 45 mm and height of 30 mm was used. The magnetic field at 5 mm distance from the surface along the cylinder axis was equal to 0.37 T [11]. A pellet formed on the wall of the tube consisting of both platelets modified with magnetite and THP-1 cells, on which platelets-FeNPs-(BSA-TRITC) adhered. The non-magnetized cell suspension was carefully removed and replaced with PBS with 2% FBS (200 μL). The pellet formed on the tube wall was then resuspended. Cell populations were analyzed both before (200 μL were taken from the experimental wells) and after magnetic separation.

Flow cytometry analysis was performed on Amnis ImageStream X Mk II (Luminex Corporation, Austin, TX, USA). Fluorescence was excited by a 405 nm laser at 20 mW power for THP-1 nuclei detection (Hoechst 33342) and 488 nm laser at 30 mW power for THP-1 (Calcein AM) and platelets (TRITC imaging). THP-1 cells with single staining were used for compensation files within subpopulation analysis by flow cytometer Flow cytometry data were processed using IDEAS software (Luminex Corporation, Austin, TX, USA). For the dataset, a population of objects with a signal in the channel corresponding to nuclear dyes was selected. The number of recorded events for each sample was 10${}^{4}$.

## 3. Results and Discussion

### 3.1. Preparation and Characterization of BSA Conjugated FeNP

**Figure 1:**
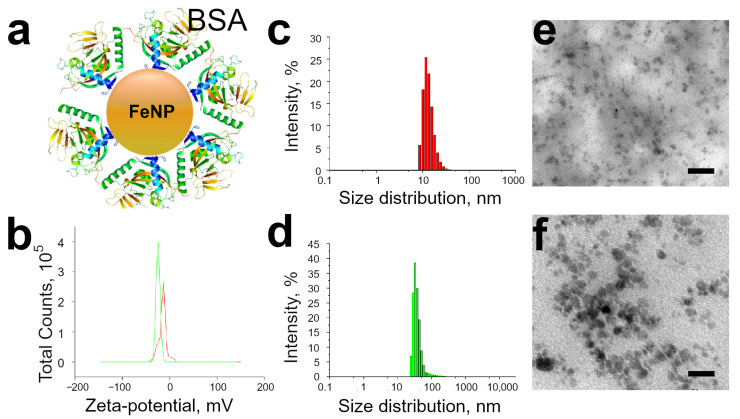
(**a**) Schematic illustration of the FeNP-BSA. (**b**) ζ-potential of non-labeled (red) and BSA-labeled (green) FeNP. Size distributions of (**c**) non-labeled and (**d**) BSA-labeled FeNP. TEM images of magnetic nanoparticles (**e**) before and (**f**) after BSA conjugation. Scale bars are 50 nm.

Magnetite nanoparticles (FeNPs) were obtained by a known protocol [31]. Before use, the magnetic particles were centrifuged to obtain particles of uniform size. The average hydrodynamic radius of FeNP before adsorption was 12.0±3 nm (Figure 1c,e), and the concentration in the solution was 1 mg/mL. The adsorption of protein molecules to the surface of FeNP leads to a triple increase in the average hydrodynamic radius of particles (33.4±7 nm, Figure 1d,f). This size of the obtained magnetite nanoparticles is optimal for capturing by platelets with the possibility of their further internalization and, therefore, the magnetic labeling of platelets [34]. According to the literature data, the conjugation of FeNP with a protein reduces cytotoxicity, improves the biocompatibility of magnetic nanoparticles, and increases the internalization of nanoparticles by platelets compared to unmodified FeNP [35,36,37,38,39,40]. Protein adsorption on the FeNP surface leads to a decrease in the ζ-potential of magnetic nanoparticles (−16.9 mV versus −23.0 mV).

### 3.2. Functionalization of Platelets by BSA Conjugated FeNP

**Figure 2:**
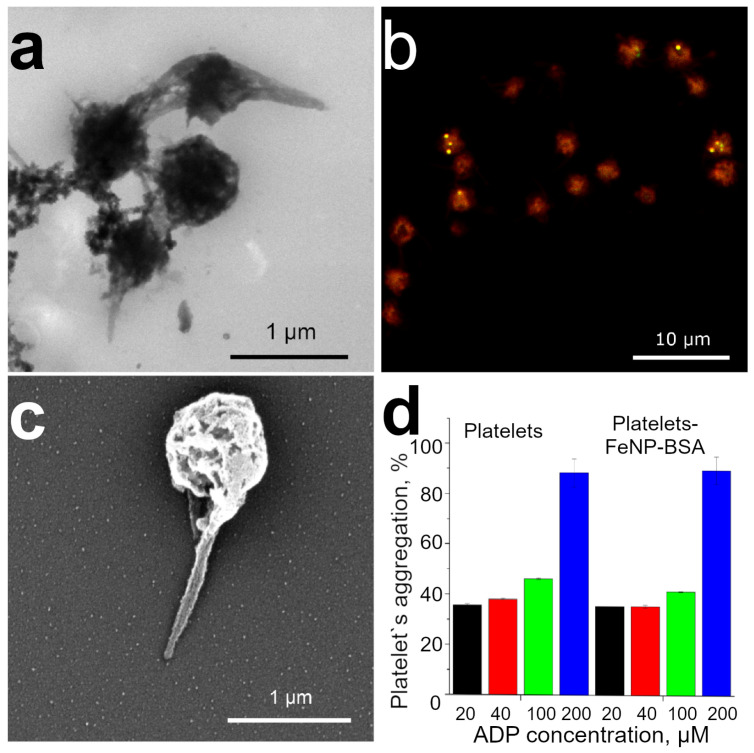
(**a**) TEM images, (**b**) CLSM, (**c**) SEM images of FeNP-BSA labeled platelets. (**d**) Platelet aggregation of non-labeled controls (platelets) and BSA-labeled (platelets-FeNP-BSA) platelets after addition of various concentrations of ADP inductor (mean ± SD, *n*= 3).

Platelets were incubated with BSA-modified magnetite nanoparticles for 30 min at 37 °C. Qualitative assessment of the internalization of FeNP by platelets was performed using TEM, CLSM, and SEM (Figure 2a–c). Figure 2a shows the high uptake of FeNP by platelets. The activation can be seen on the TEM image for only the single platelet; it turns into a spherical state and releases long processes. The formation of platelet aggregates occurs mainly not due to their activation, but due predominantly to the magnetic interaction between BSA-modified FeNPs. CLSM images show platelet aggregates containing FeNP-BSA(FITC) (Figure 2b). The FeNP surface was modified with a fluorescently labeled BSA(FITC) protein, and platelets were labeled with Nile Red fluorescent membrane dye. Morphological platelet changes after FeNP-BSA labeling were demonstrated using SEM microscopy (Figure 2c).

The functional capacity of platelets directly depends on the degree of their aggregation (Figure 2d). Non-activated platelets are able to perform their protective functions. When interacting with various inducers, platelets are activated, forming aggregates, and, as a result, they lose their further functional ability [38]. The degree of platelet activation and dysfunction after internalization of FeNP-BSA was assessed by the spectrophotometric method according to the light transmission of platelets. ADP (30 μL, 20–200 μM) was used as an inducer of platelet aggregation, native platelets incubated in the HEPES-Tyrode buffer served as a negative control. The decrease in turbidity with an increase in platelet aggregation was determined by measuring the optical density at 650 nm. An increase in the amount of ADP added to platelets led to a dependent aggregation of the latter on the concentration of the agonist [41]. According to the data of spectrophotometric analysis, platelet labeling with magnetic nanoparticles modified by BSA did not cause significant platelet activation (Figure 2d) in comparison to control non-modified platelets. The obtained data are consistent with the results of early studies on the preservation of nanoparticle-induced platelet aggregation activity [35,42,43]. The addition of a small ADP amount (20, 40 μM) leads to an initial change in the shape of platelets, accompanied by primary aggregation. ADP-induced aggregation promotes a secondary wave of aggregation due to the release of synthesized ADP, serotonin, and fibrinogen from platelet granules [44]. The subsequent secondary wave of platelet aggregation is irreversible. The presence of the irreversible two-wave aggregation of FeNP-BSA labeled platelets indicates the preservation of the functional ability of the cells. The addition of high concentrations of ADP (100, 200 μM) leads to simultaneous primary and secondary aggregation waves, which are accompanied by an increase in the number of platelet aggregates.

### 3.3. Platelet Decoration by BSA Conjugated FeNP

**Figure 3:**
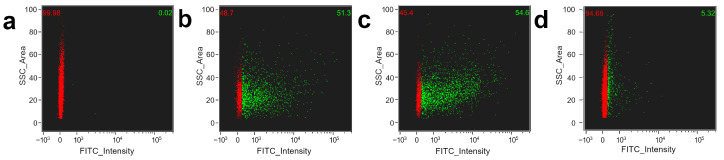
Changes in the platelet population after incubation with BSA-FITC conjugated FeNP observed using a flow cytometer. (**a**) A population of unstained platelets, showing the absence of autofluorescence in the area characteristic to the FITC dye. (**b**) Platelets after 30 min of incubation with magnetite nanoparticles. (**c**) Cells that have undergone a magnetic separation procedure. (**d**) The cell population remaining in the washing effluent. Red and green numbers show the percentage of platelets without and with FeNP, respectively.

The efficiency of platelet functionalization with magnetite nanoparticles conjugated with FITC was observed using flow cytometry. Before incubation, the autofluorescence of platelets at wavelengths of 480–560 nm was not detected (Figure 3a). However, 30 min adsorption of FeNP conjugated with FITC led to the population dividing into cells that have and did not have a fluorescent signal corresponding to FITC (Figure 3b). The proportion of fluorescent platelets increases after using the OctoMACS Separator (Figure 3c). In contrast, the population of FITC-labeled cells is extremely low in the washing effluent (Figure 3d). Thus, the proposed method for the additional functionalization of platelets is able to provide a large population of cells with adsorbed magnetite nanoparticles. There are a number of articles that report the successful incorporation of ferucarbotran magnetic nanoparticles contained in Resovist into platelets [38,45]. Resovist nanoparticles are superparamagnetic iron oxide, consisting of magnetite (Fe_3_O_4_) and maghemite (γ-Fe_2_O_3_) polycrystals coated by a carboxydextran [46]. The resultant diameter is 62 nm. Resovist is usually applied for diagnostic purpose (MRI) but, as we suppose, the polycrystalline that consists of magnetite nanoparticles only will be able to provide a precise navigation and retention in specific location by the gradient of magnetic force because of better magnetization compared to maghemite [47]. Alejandro G. Roca et al. showed that the saturation magnetization is greater for magnetite than for maghemite [48]. Additionally, an increase in the size of nanoparticles leads to an increase in both the saturation magnetization and coercivity. An example of the effective motion control of polymeric microcapsules inlaid with magnetite has been shown previously [11]. We believe that the proposed method for the functionalization of platelets with nanoparticles with an average size of 33 nm will provide effective control of their movement under the external magnetic field.

### 3.4. Platelets Interaction with Cell of Different Origin

**Figure 4:**
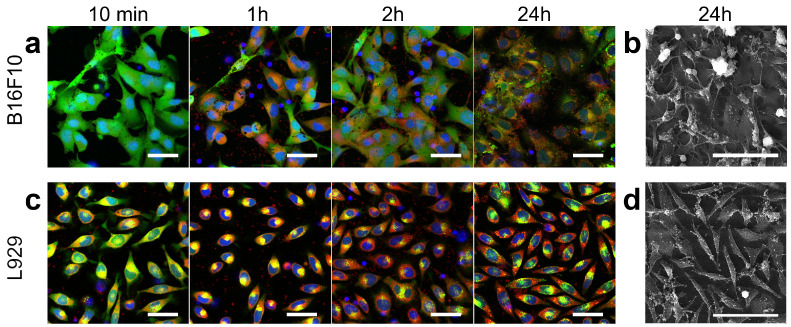
Microscopy of adherent cell cultures incubated with platelets for 24 h. Fluorescence microscopy of B16F10 (**a**) and L-929 (**c**) during incubation with platelets. Blue, green, and red colors correspond to Hoechst (nucleus), Calcein AM (cytoplasm), and Nile Red (preliminary staining of isolated platelet membrane) staining, respectively. All cells were stained before adding platelets and no restaining was performed 24 h later. Scale bars are 20 μm. Scanning electron microscopy of (**b**) B16F10 and (**d**) L-929 after 24 h of incubation with platelets. Scale bars are 100 μm.

The interaction of platelets that underwent the magnetic separation procedure with adhesive cancerous or non-cancerous cell lines was observed (Figure 4). After the addition, platelets are distributed throughout the volume of the culture medium and only single platelets are visible at the bottom. However, the majority of the platelets sediment after an hour. Fluorescence images of control cells without platelets addition are shown in Figure S1.

Similar trends were found for both cell lines. First, during the first hour after the platelets were added, the cells began to decrease the area of adhesion to the bottom of the Petri dish (round up). However, the cells returned to an elongated and spread-eagled form by 2 h. Secondly, there was an increase in the number of cells after 24 h of incubation with platelets, which indicates the maintenance of the proliferative activity of B16F10 and L-929. Thirdly, platelets accumulated mainly on cell membranes by 24 h, while many platelets were in the space between cells in the first hours, which was confirmed by both fluorescence and electron microscopy. Additionally, using scanning electron microscopy, it was shown that platelet degranulation [49] and fibrin formation [50] occurred after 24 h of co-cultivation (Figure 4b,d, Figures S2 and S3). Finally, the process of Nile Red dye transition from platelets to the membranes of adherent cells was clearly visible. Such a process can be considered the transfer of a certain model substance during the delivery of biologically active molecules [29]. However, it should be noted that the transfer process varied in different cell lines. Thus, for example, L-929 stained more Nile Red than B16F10 during the observation time. This effect is probably directly related to the different structure and functions of cells. The B16F10 cell line is the result of the malignant degeneration of melanocytes, the function of which is the synthesis of melanin granules. As is known, melanin is capable of quenching the fluorescent signal of a dye, due to which a lower intensity of cell luminescence is visually observed compared to cells without melanin (L-929) [51]. Another cancer cell line, namely CT26 (murine colorectal carcinoma), was considered to rule out the effect of melanin on the observed fluorescence intensity from Nile Red dye (Figure S4). Visually, the transfer of the lipophilic dye to the CT26 membrane is efficient. Already after 3 h of co-cultivation, a significant number of CT26 cells had a membrane stained with Nile Red. Thus, the possibility of model substance delivery using platelets was qualitatively demonstrated. However, the considered method of delivery is not selective. This problem can be solved by using molecules during platelet loading, to which cancerous cells are more sensitive compared to normal cells. Examples are the bacterial ribonucleases barnase and binase [52,53].

**Figure 5:**
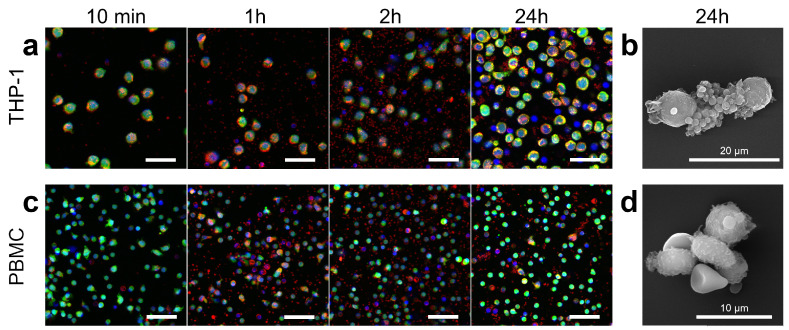
Microscopy of suspension cell cultures incubated with platelets for 24 h. Fluorescence microscopy of THP-1 (**a**) and PBMC (**c**), during incubation with platelets. Blue, green, and red colors correspond to Hoechst (nucleus), Calcein AM (cytoplasm), and Nile Red (preliminary staining of isolated platelet membrane) staining, respectively. All cells were stained before adding platelets and no restaining was performed 24 h later. Scale bars are 20 μm. Scanning electron microscopy of THP-1 (**b**) and PBMC (**d**) after 24 h of incubation with platelets. Scale bars are 100 μm.

Further, the interaction with the suspension cell lines was considered. THP-1 was used as a continuous cell line and model of blood cancer (cancer cells circulating in the bloodstream) and, in addition, peripheral blood mononuclear cells (PBMCs) obtained from the same blood as platelets were implemented. Trends similar to adherent cultures were found. These are the transfer of lipid dye from platelets to monocyte cells and the preservation of proliferation in THP-1 cells (Figure 5 and Figure S5). However, there were a number of dissimilarities in the processes involving THP-1 and PBMC cells. Platelet–platelet and platelet–mononuclear cell aggregation was observed when platelets were incubated with THP-1, while in the case of PBMC, only platelet–platelet aggregation occurred (Figure S6). Scanning electron microscopy also confirms the presence of a large number of platelets adhere to THP-1 (Figure 5b and Figure S7), although it is worth noting that this method may present some artifacts due to the large number of repetitions of centrifugation during dehydration procedures, which contributes to the cells coalescing in pellet.

The absence of aggregation between platelets and PBMC indicates their weak activation during the BSA-conjugated FeNP functionalization procedure, while a slight increase in the concentration of lysophosphatidic acid in the blood leads to platelet–monocyte aggregate formation as reported [54]. In general, the aggregation between platelets and white blood cells is considered a sign of the development of various pathological processes: atherosclerosis, inflammation, vascular damage, and infection [55,56]. In this regard, the lack of aggregation stimulation after the proposed method of platelet functionalization is considered a positive and promising feature for the further design of therapeutic and diagnostic systems based on them.

### 3.5. Controlling Cell Movement after Platelet Adherence

Despite the synergy of cancer cells and platelets in the progression of cancer described in the literature [57,58], there is currently an opinion that the use of platelets in anticancer therapy can open up new possibilities [59]. Indeed, we observed adhesion and transfer of a model low molecular weight lipophilic substance from platelets to other cells, which can be considered as targeted drug delivery and the ability to navigate the cells with absorbed magnetite nanoparticles for cancer cells elimination from the bloodstream.

**Figure 6:**
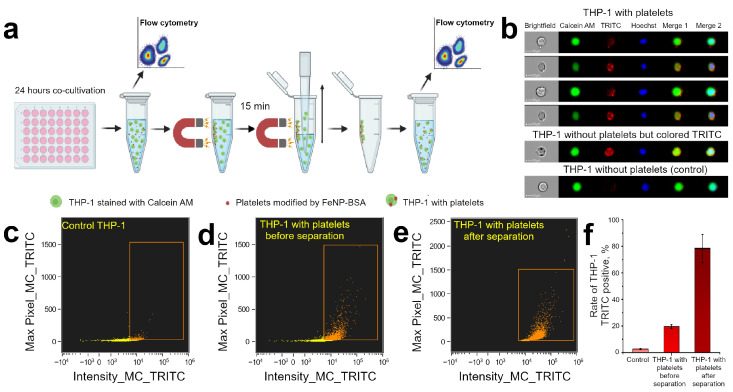
(**a**) Schematic representation of the experimental procedure in study of THP-1 cells subpopulations during co-cultivation with platelets modified with magnetite nanoparticles. (**b**) Examples of THP-1 in cell population (Calcein AM channel corresponds to THP-1 cytoplasm, TRITC channel corresponds to platelets, Hoechst stained the THP-1 nuclei, Merge 1 is overlapping of Calcein AM and TRITC channels, Merge 2 joined all fluorescence channels). (**c**–**e**) Bivariate scatterplots defined total TRITC intensity and presence of bright spots in (**c**) control samples without platelets co-cultivation, (**d**) experimental sample with co-cultivation before and (**e**) after magnetic separation. The boxed area defines a population with intense TRITC fluorescence provided by exogenous dyes characteristic for platelets in this experiment. (**f**) Percentage of THP-1 associated with TRITC fluorescence.

Cell populations were analyzed before and after exposure to a magnetic field to demonstrate the ability to control the movement of THP-1 cells after interaction with functionalized platelets. The sequence of actions is shown in Figure 6a. The co-cultivation of THP-1 and platelets lasted 24 h. The test samples were then transferred from the wells of the 48-well plate to a 1.5 mL tube. A small portion of the sample was taken for flow cytometer measurements. The remaining part was exposed to a NdFeB magnet for 15 min. During this time, a pellet formed on the wall of the test tube. All of the liquid with cells not attached to the walls was carefully removed from the test tube in the presence of a magnetic field. Then PBS with 2% FBS was added to the tube. The pellet was gently resuspended and analyzed with a flow cytometer.

The gating strategy is shown in Figure S8. Cells of different outlook were present in the considered populations (Figure 6b). Cells within or close to the surface of which platelets were found have bright dots of fluorescence in the TRITC channel. At the same time, some of the cells had uniform strong TRITC staining. This phenomenon may be associated with the detachment of the THP-1 cell and platelet due to mechanical action from other cells when the sample was transferred to a 1.5 mL tube or suspended after removal of non-magnetized cells. We considered such cells as interacting with platelets and, accordingly, were taken into account when calculating the proportion of THP-1 TRITC-positive. Cells lacking fluorescence in the TRITC channel were considered as not interacting with platelets.

A very small part of the cells had a strong TRITC fluorescence in the control sample (Figure 6c) and their proportion did not exceed 3% (Figure 6f). TRITC-positive became 20% of the tested cells after 24 h of co-incubation THP-1 and platelets (Figure 6d). It means that this was the proportion of cells to which platelets functionalized with FeNP-(BSA-TRITC) adhered, and the movement of which, as we believe, could be controlled using a magnetic field. Analysis of cell populations after magnetic retention on the tube wall showed that almost 80% of the cells were shown TRITC fluorescence (Figure 6e). Thus, the population of cells that did not interact with platelets was almost completely removed from the examined sample. An assessment of the total number of cells in the sample before and after magnetic separation was also made (Table S1), which confirmed that after magnetic localization, about 20% of the initial number of cells remained in the sample.

Currently, the question of the effectiveness and safety of magnetically assisted hemodialysis is being considered [60,61]. While individual molecules identify as targets for trapping and removal from the blood, cancer cells corresponding to both myeloid cancer and metastatic cancer cells can be cleared from the bloodstream by FeNPs-functionalized platelets. In the presented study, THP-1 suspension cells were the prototype of the cells that should be eliminated from the blood vessels. It seems possible to increase the proportion of cells to which platelets bind through the use of vector molecules (antibodies, aptamers, or DARPin). In this work, we showed the fundamental possibility of controlling the movement of a cell in suspension after platelet-FeNP adheres to it.

## 4. Conclusions

It is well-known that platelets actively participate and help cancer cells in the process of tumor growth and metastasis. In this regard, it seems rational to consider platelets as carriers, for example, of anticancer drugs. The granting of platelets with the ability to control movement under the influence of external forces further expands their scope. In this work, the surface of magnetic nanoparticles (FeNP) was modified with bovine serum albumin (BSA). The size of the resultant FeNPs has increased more than 2 times (from 12±3 nm to 33±7 nm. The ζ-potential of the FeNP surface was reduced, which contributed to their efficient incorporation into platelets. BSA-conjugated FeNP-modified platelets were imaged by TEM and CLSM, and platelet populations were analyzed before and after magnetic particle adsorption. The study of platelet aggregation with the addition of an ADP inductor showed that the considered functionalization does not lead to the significant activation of platelets, and therefore preserves their functional ability. In vitro studies have confirmed that modified platelets are able to adhere to cancerous and foreign cells, while they do not interact with cells derived from the same blood. The transition of a lipophilic dye (model substance) from platelets to co-cultivation cells was also clearly demonstrated. The magnetic separation of cells with platelets in the tube was shown. Thus, the proposed approach opens the avenue both in the delivery of therapeutic agents and for the magnetic interaction with platelets and cancer cells with attached platelets.

## Data Availability

The data presented in this study are available on request from the corresponding author.

## Supplementary Materials

The following supporting information can be downloaded at: https://www.mdpi.com/article/10.3390/pharmaceutics15010214/s1, Figure S1: Fluorescence microscopy of control B16F10 (**a**,**b**) and L-929 (**c**,**d**) at 1st and 2nd day of observation without co-cultivation with platelets. Blue color corresponds to Hoechst (nucleus) staining, green—Calcein AM (cytoplasm), red—Nile Red (preliminary staining of isolated platelet membrane). Scale bar is 20 μm; Figure S2: Scanning electron microscopy of B16F10 (**a**) and L-929 (**b**) after 24 h of incubation with platelets. Scale bar is 5 μm; Figure S3: Scanning electron microscopy of control B16F10 (**a**) and L-929 (**b**) without co-cultivation with platelets. Scale bar is 20 μm; Figure S4: Fluorescence microscopy of CT26 at 1st and 2nd day of observation with co-cultivation with platelets. Blue, green, and red colors correspond to Hoechst (nucleus), Calcein AM (cytoplasm), and Nile Red (preliminary staining of isolated platelet membrane) staining, respectively; Figure S5: Fluorescence microscopy of control THP-1 (**a**,**b**) and PBMC (**c**,**d**) at 1st and 2nd day of observation without co-cultivation with platelets. Blue and green colors correspond to Hoechst (nucleus) and Calcein AM (cytoplasm) staining, respectively. Scale bar is 20 μm; Figure S6: Fluorescence microscopy of THP-1 (**a**) and PBMC (**b**) after 24 h of incubation with platelets. Blue, green, and red colors correspond to Hoechst (nucleus), Calcein AM (cytoplasm), and Nile Red (preliminary staining of isolated platelet membrane) staining, respectively. Scale bar is 20 μm; Figure S7: Scanning electron microscopy of control THP-1 (**a**) and PBMC (**b**) without co-cultivation with platelets. Scale bar is 10 μm; Figure S8: Gating strategy for calculating rate of THP-1 associated with TRITC dye. The R1 population consisted of well-focused cells. Singlets were chosen in the R2 population. The R3 population provided the objects with good nucleus and Calcein AM staining. The R4 population presented cells with fluorescence intensity in the TRITC channel more than autofluorescence. R5 population is a population with internalized platelets. The described protocol is standard. Internalization score is determined by detecting fluorescently labeled particles within a mask outlining the cell surface, based on a phase contrast image of the cell [60,62]; Table S1: Counting the number of cells in the sample and the percentage of cells retained by the magnetic field. Values are presented as mean ± SD. Number of measurements 3.
