# Supplementary material for: Magnetic Platelets as a Platform for Drug Delivery and Cell Trapping
Source: Pharmaceutics. 2023 Jan 7;15(1):214. doi: 10.3390/pharmaceutics15010214 (PMC9866132; doi:10.3390/pharmaceutics15010214)
Supplement: Supplementary file 1 [file pharmaceutics-15-00214-s001.zip › pharmaceutics-2103831-supplementary.pdf]

Magnetic Platelets as a Platform for Drug Delivery and Cell Trapping

Oksana A. Mayorova, Olga I. Gusliakova, Ekaterina S. Prikhozhdenko, Roman A. Verkhovskii and Daniil N. Bratashov

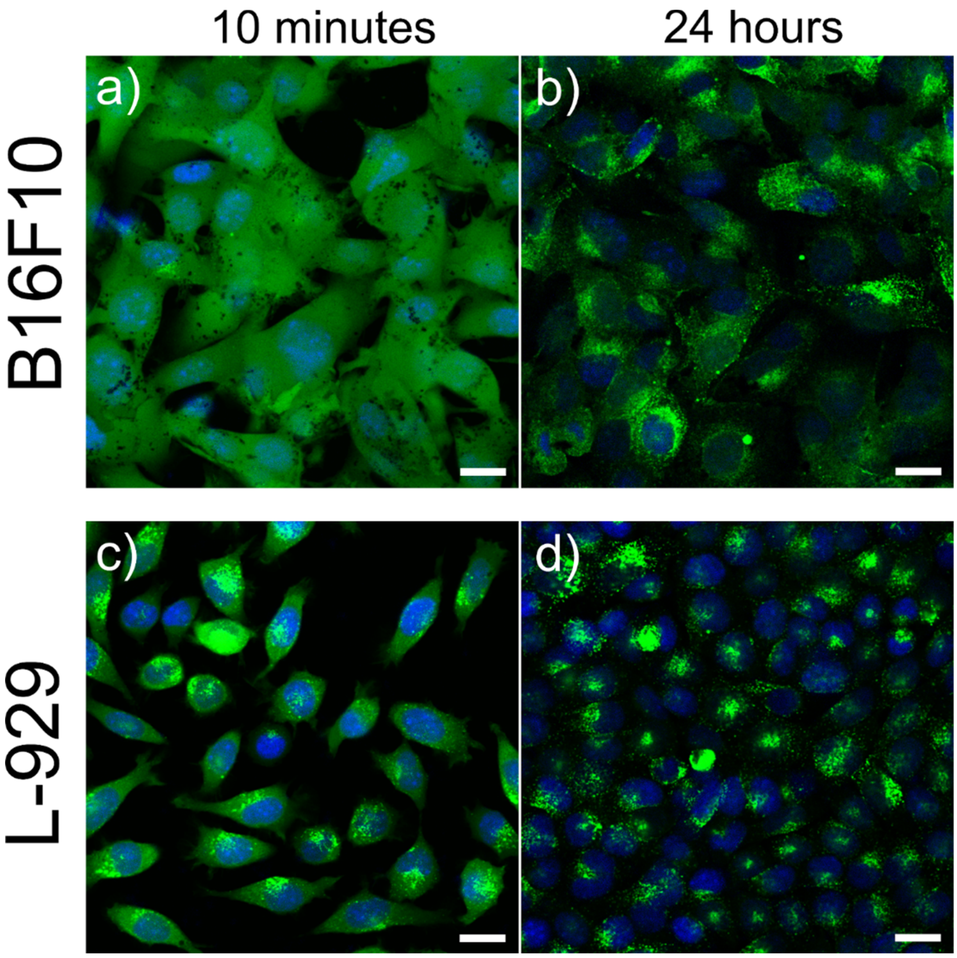

Figure S1. Fluorescence microscopy of control B16F10 (a, b) and L-929 (c, d) at 1st and 2nd day of observation without co-cultivation with platelets. Blue color corresponds to Hoechst (nucleus) staining, green - Calcein AM (cytoplasm), red - Nile Red (preliminary staining of isolated platelet membrane). Scale bar is 20 μm.

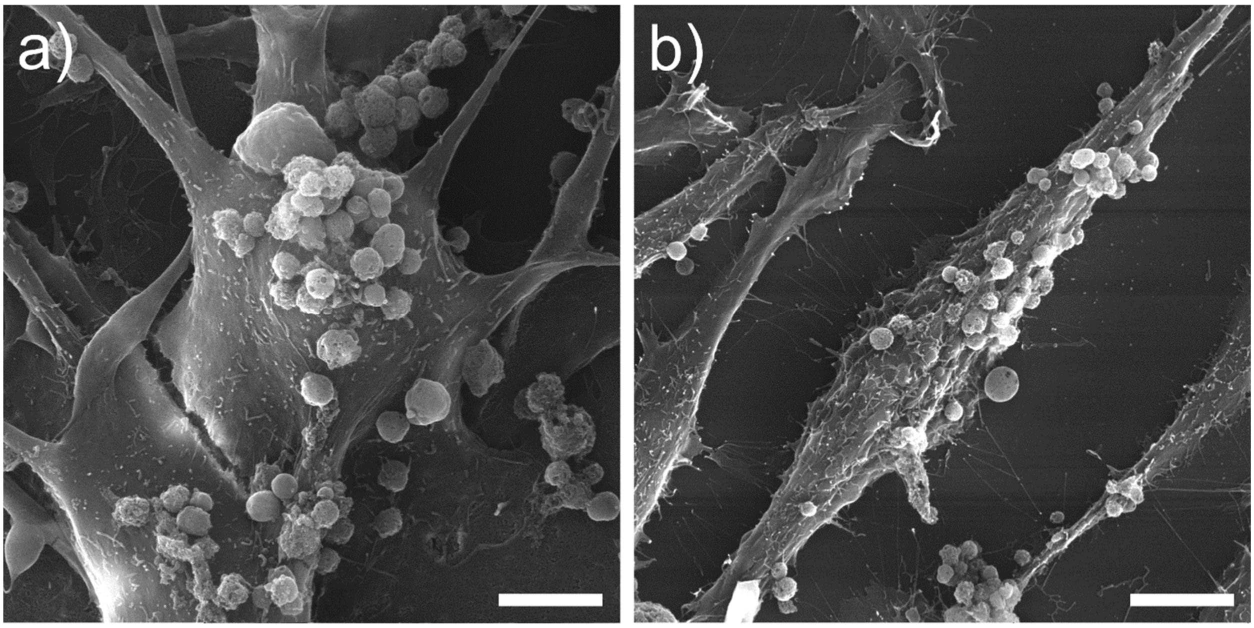

Figure S2. Scanning electron microscopy of B16F10 (a) and L-929 (b) after 24 hours of incubation with platelets. Scale bar is 5 μm.

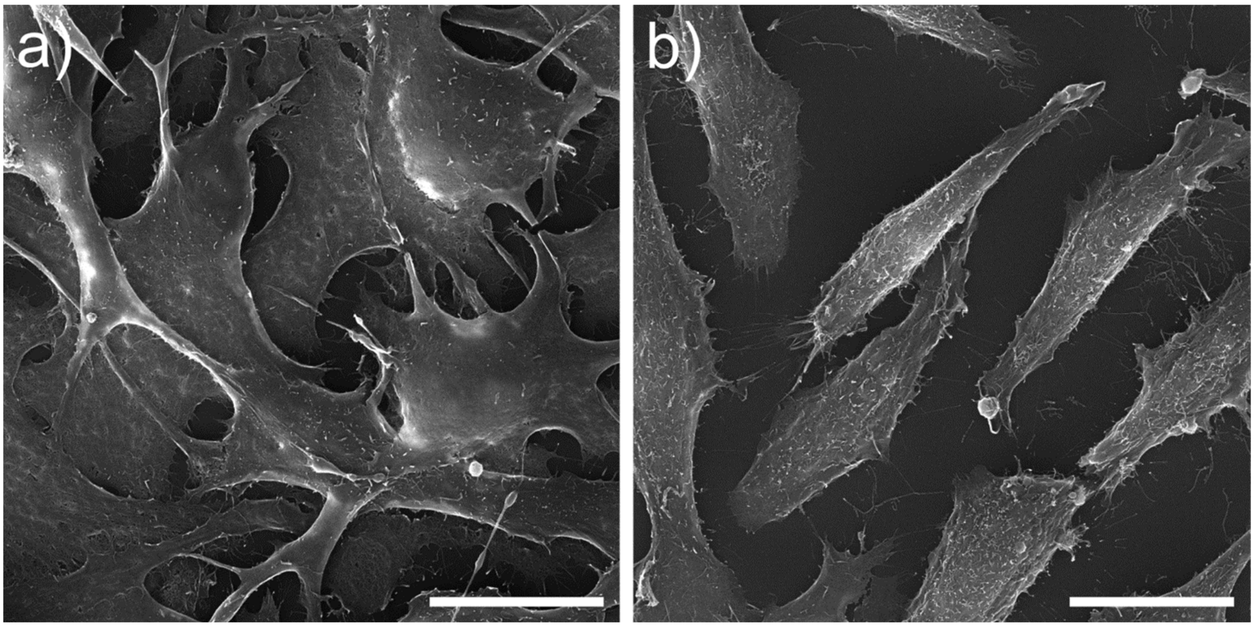

Figure S3. Scanning electron microscopy of control B16F10 (a) and L-929 (b) without co-cultivation with platelets. Scale bar is 20 μm.

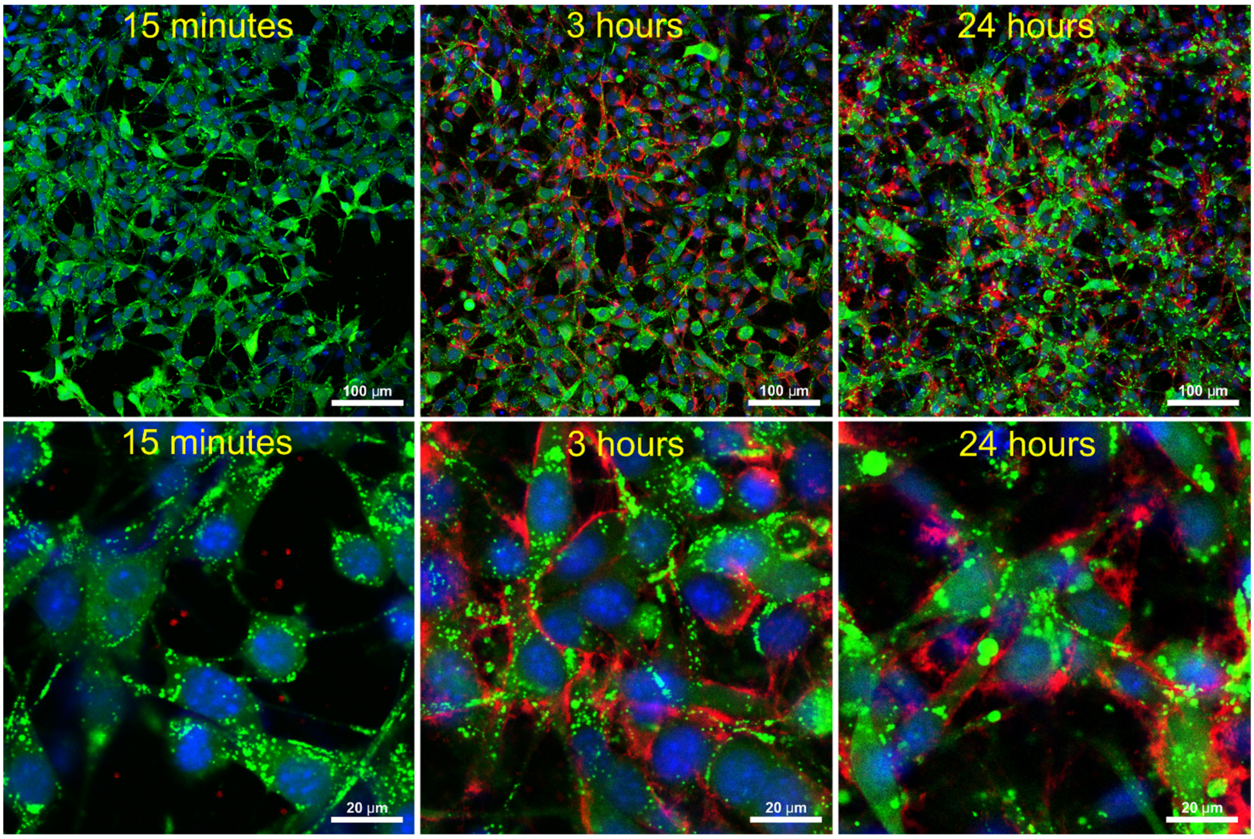

Figure S4. Fluorescence microscopy of CT26 at 1st and 2nd day of observation with co-cultivation with platelets. Blue, green, and red colors correspond to Hoechst (nucleus), Calcein AM (cytoplasm), and Nile Red (preliminary staining of isolated platelet membrane) staining, respectively.

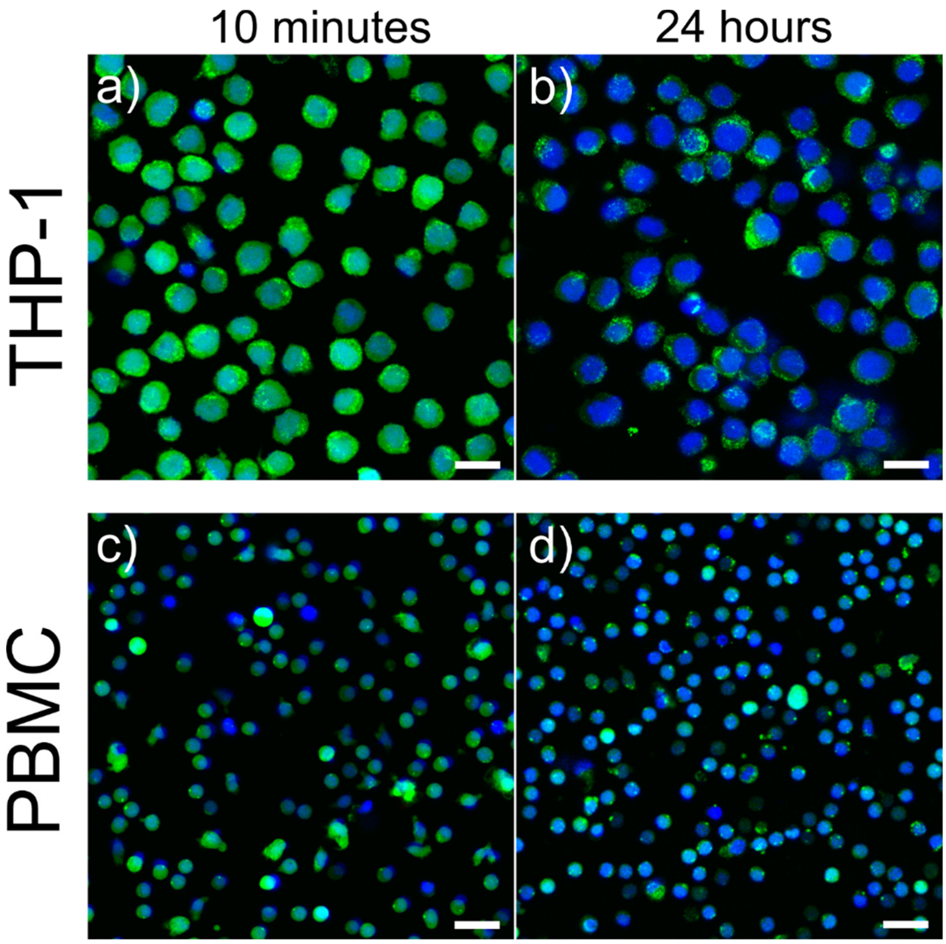

Figure S5. Fluorescence microscopy of control THP-1 (a, b) and PBMC (c, d) at 1st and 2nd day of observation without co-cultivation with platelets. Blue and green colors correspond to Hoechst (nucleus) and Calcein AM (cytoplasm) staining, respectively. Scale bar is 20 μm.

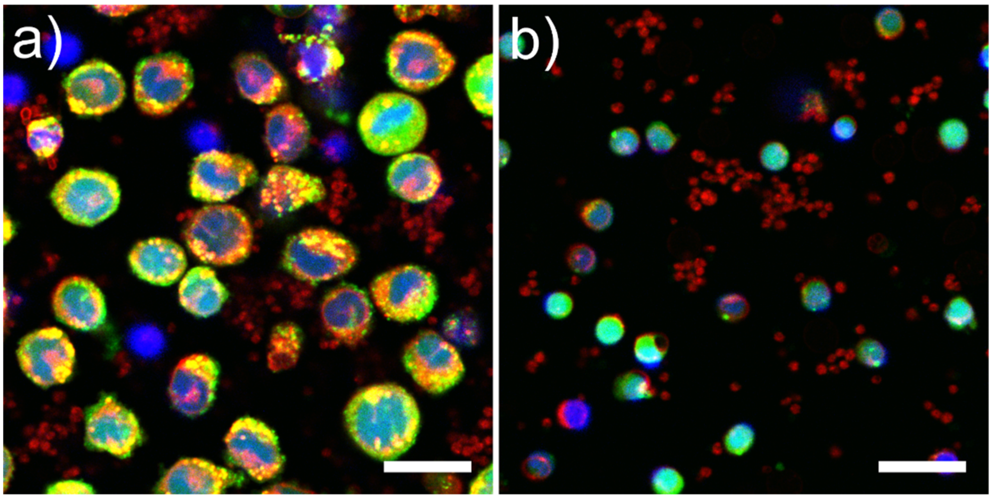

Figure S6. Fluorescence microscopy of THP-1 (a) and PBMC (b) after 24 hours of incubation with platelets. Blue, green, and red colors correspond to Hoechst (nucleus), Calcein AM (cytoplasm), and Nile Red (preliminary staining of isolated platelet membrane) staining, respectively. Scale bar is 20 μm.

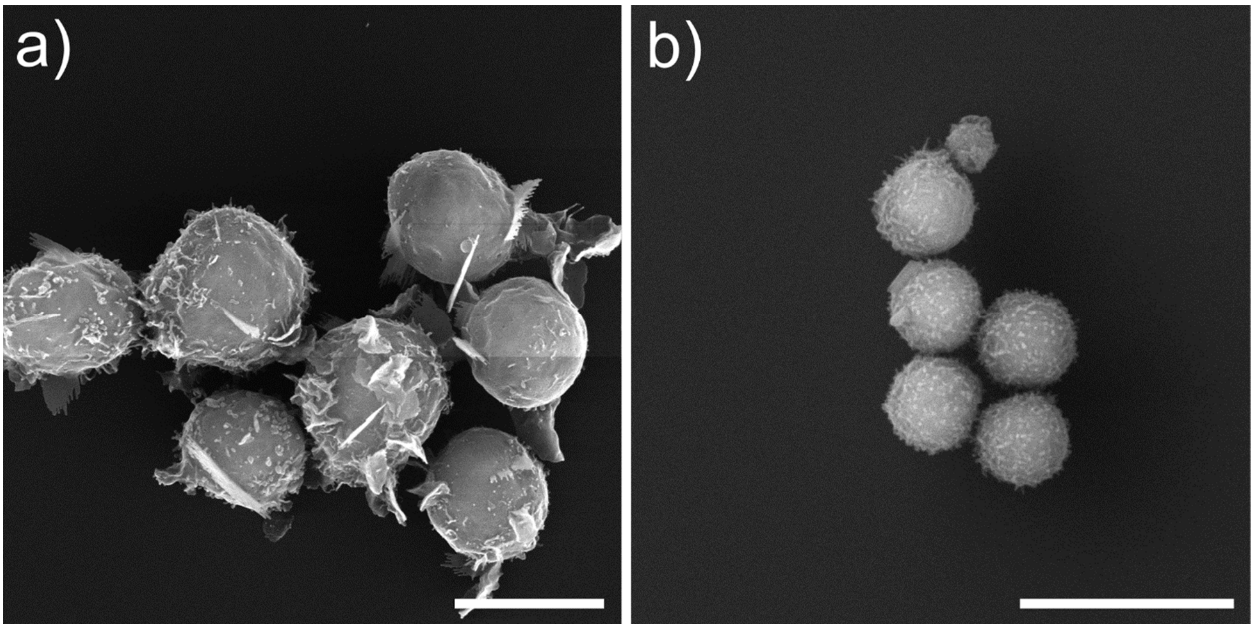

Figure S7. Scanning electron microscopy of control THP-1 (a) and PBMC (b) without co-cultivation with platelets. Scale bar is 10 μm.

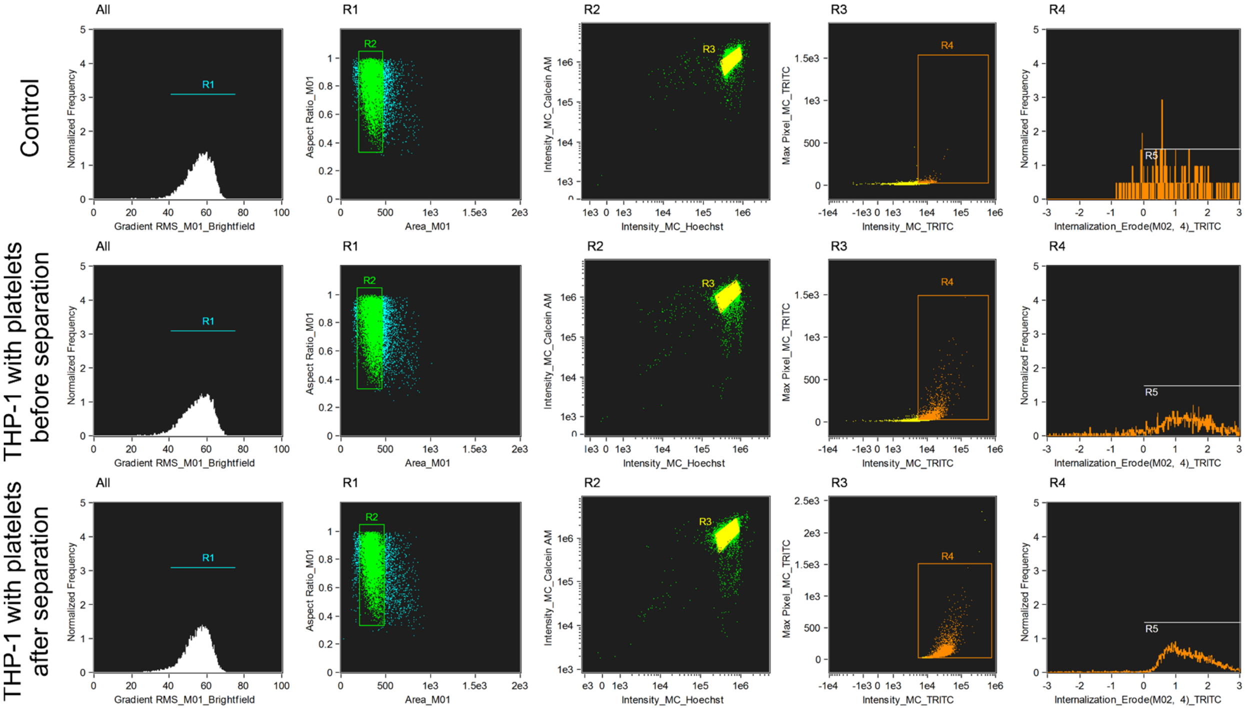

Figure S8. Gating strategy for calculating rate of THP-1 associated with TRITC dye. The R1 population consisted of well-focused cells. Singlets were chosen in the R2 population. The R3 population provided the objects with good nucleus and Calcein AM staining. The R4 population presented cells with fluorescence intensity in the TRITC channel more than autofluorescence. R5 population is a population with internalized platelets. The described protocol is standard. Internalization score is determined by detecting fluorescently labeled particles within a mask outlining the cell surface, based on a phase contrast image of the cell [1,2].

Table S1. Counting the number of cells in the sample and the percentage of cells retained by the magnetic field. Values are presented as mean ± SD. Number of measurements 3.

Number of cells in a sample **before** magnetic separation	Number of cells in a sample **after** magnetic separation	Percentage of cells retained by the magnetic field
$(1.40 \pm 0.03) \times 10^6$	$(0.30 \pm 0.03) \times 10^6$	21.7%±1.3%

References:

1. Phanse, Y.; Ramer-Tait, A.E.; Friend, S.L.; Carrillo-Conde, B.; Lueth, P.; Oster, C.J.; Phillips, G.J.; Narasimhan, B.; Wannemuehler, M.J.; Bellaire, B.H. Analyzing Cellular Internalization of Nanoparticles and Bacteria by Multi-spectral Imaging Flow Cytometry. Journal of Visualized Experiments 2012, 64, e3884 https://doi.org/10.3791/3884.

2. Ploppa, A.; George, T.C.; Unertl, K.E.; Nohe, B.; Durieux, M.E. ImageStream cytometry extends the analysis of phagocytosis and oxidative burst. Scandinavian Journal of Clinical and Laboratory Investigation 2011, 71, 362–369. https://doi.org/10.3109/00365513.2011.572182.
